# Low expression of long noncoding RNA MT1JP is associated with poor overall survival in gastric cancer patients: Protocol for meta-analysis

**DOI:** 10.1097/MD.0000000000010394

**Published:** 2018-05-25

**Authors:** Chenglou Zhu, Jichun Ma, Yaoqi Li, Yongbin Zhang, Mingxu Da

**Affiliations:** a College of Clinical Medicine, Gansu University of Chinese Medicine; b Department of General Surgery Intensive Care Unit, Lanzhou University Second Hospital, Lanzhou; c College of Clinical Medicine, Ningxia Medical University, Yinchuan; d Department of Surgical Oncology, Gansu Provincial People's Hospital, Lanzhou, China.

**Keywords:** gastric cancer, long noncoding RNA, meta-analysis, overall survival

## Abstract

**Background:**

Although several researches have investigated the association between development and metastasis of gastric cancer (GC) and the expression level of MT1JP, there are no consensuses about whether its expression is associated with overall survival (OS) and clinical feature for GC patients.

**Methods:**

The databases including PubMed, EMBase databases, and the Cochrane Library were searched from inception to January 30, 2016, to identify the eligible studies. The quality of included studies was assessed according to reporting recommendations for tumor marker prognostic studies (REMARK). The association between expression level of LncRNA HOTAIR with OS for GC patients was assessed by calculating the pooled hazard ratio (HR) and 95% confidence interval (95% CI) using STATA version 12.0. Heterogeneity among studies will be assessed using the *I*^2^ statistic.

**Results:**

Randomized controlled trials (RCTs), prospective cohort studies, and case–control studies will be used for the qualitative and quantitative synthesis of the meta-analysis to explore the association between MT1JP expression levels with OS for gastric cancer patients.

**Conclusion:**

We aim to draw an objective conclusion of the association between MT1JP expression levels with OS for gastric cancer patients.

## 1 Introduction

Despite its overall declining prevalence, gastric cancer (GC) is still the third leading cause of cancer-related death and remains the fourth most common malignancy in Eastern Asia, especially in China.^[1,2]^ About a million GC patients were diagnosed and over a number of 700,000 persons died from GC in 2012.^[3]^ GC is a main contributor of disability-adjusted life-years to the global burden following lung cancer and liver carcinoma and account for 20%, 23%, and 28%.^[4]^ Owing to lack of the specific biomakers at the early stage, growing number of GC patients were diagnosed in advanced stage, making it a short overall survival (OS) for GC patients.^[5]^

Long noncoding RNA (LncRNA) is a type of transcribed RNA molecules, which ranged from 200 to 100 kbp in length.^[6]^ Due to its involvement in lots of biological processes such as imprinting,^[7]^ embryogenesis,^[8]^ transcriptional regulation,^[9]^ and epigenetic regulation.^[10]^ Therefore, it plays a crucial role in the growth and development of the human body. However, whether its expression correlated with OS and clinical feature for GC patients remains inconclusive and no consensus has been achieved. Liu et al^[11]^ identified a lncRNA MT1JP, which appears to play a significant role in the inhibition of tumors. Their data showed that MT1JP has lower expression in tumor tissue samples than in matched normal tissues. Therefore, a meta-analysis was conducted to get an overall understanding of LncRNA MT1JP in evaluating OS and its clinical characteristics for gastric cancer patient.

## 2 Materials and methods

This protocol for meta-analysis is performed according to the Preferred Reporting Items for Systematic Review and Meta-analysis Protocols (PRISMA-P) statement.^[12]^

### 2.1 Search strategy

To identify all publications relevant to the association between LncRNA HOTAIR and OS of GC patients, we performed the comprehensive literature search using the PubMed, EMBASE database, and the Cochrane Library from inception to January 30, 2018. The search terms were following: “gastric cancer” OR “stomach neoplasm” OR “stomach cancer” OR “gastric neoplasm” OR “gastric carcinoma” OR “stomach carcinoma” AND “Long Non-coding RNA” OR “Long Noncoding RNA” OR “Long Non Coding RNA” OR “LncRNA.” The references of included studies and related systematic reviews were tracked. Our research was limited to English-language article, but not to time period.

2.1.1 Search strategy in PubMed

#1 “Long Non-coding RNA”[Title/Abstract] OR “Long Noncoding RNA”[Title/Abstract] OR “Long Non Coding RNA”[Title/Abstract] OR “LncRNA”[Title/Abstract]

#2 “RNA, Long Noncoding”[Mesh]

#3 “Gastric cancer∗”[Title/Abstract] OR “Gastric Neoplasm∗”[Title/Abstract] OR “Gastric Carcinoma∗”[Title/Abstract] OR “Stomach Cancer∗”[Title/Abstract] OR “Stomach Neoplasm∗”[Title/Abstract] OR “Stomach Carcinoma∗”[Title/Abstract]

#4 “Stomach Neoplasms”[Mesh]

#5 #1 OR #2

#6 #3 OR #4

#7 #5 AND #6

### 2.2 Study design

Randomized controlled trials (RCTs), prospective cohort studies, and propensity-matched comparative studies, and case–control studies will be used for the qualitative and quantitative synthesis of the meta-analysis.

### 2.3 Selection criteria

Eligible studies for this meta-analysis met the following criteria: patients were confirmed GC by pathological or histological examination; explored LncRNA expression in GC subjects; evaluation of the association between HOTAIR and OS; sufficient data to estimate hazard ratio (HR) and its 95% confidence interval (95% CI); and publication as a full article in English.

The exclusion criteria were studies assessing the molecular structure and function of HOTAIR; review articles, case reports, abstracts, editorials, letters, and meta-analysis; the article without sufficient data to analysis after contacting study authors; and duplicate publications.

### 2.4 Data extraction

Two reviewers independently extracted relevant data from the included studies by using the per-designed data form. Any disagreements were resolved by discussion. Data retrieved from each publication included basic characteristics of each studies such as the first author, year of publication, country, sample size, control sources, level of LncRNA expression, cut-off values; OS: if the HRs were reported in the publication, it were obtained from reading text directly. Otherwise, we recalculated the HRs from the published data including the number of GC patients at risk or its *P* value. However, if those were not possible, we attempted to obtain the HRs from Kaplan–Meier curves by using the HR digitizer software Engauge 4.0 and software GetData Graph Digitizer 2.24 to extract and digitize the survival data.^[13]^ The specific method is to import clear and interpretable pictures from the PDF, draw points and extract data in the Engauge Digitizer 4.1 software, and finally calculate the HRs estimates based on the obtained data.

### 2.5 Quality assessment

Quality assessment for each eligible study was carried out by the same 2 reviewers who read and scored each publication independently according to reporting recommendations for tumor marker prognostic studies (REMARK).^[14]^ The REMARK checklist was categorized into 4 main dimensions and 20 items: introduction, materials and methods (patients, specimen characteristics, assay methods, study design, and statistical analysis methods), results (data, analysis and presentation), and discussion. Each item was answered to “reported,” “unclear,” or “not reported” and given a score “1,” “0.5,” or “0.” Finally, its total score of each study represented the result of quality assessment.

### 2.6 Statistical analysis

The strength of association between the expression level of LncRNA HOTAIR and OS for GC patients was assessed by calculating the pooled HR and 95% CI by using software STATA 12.0. Heterogeneity assumption was evaluated with a χ^2^-based Q-test: if the *P* value was more than .1 or *I*^2^ was less than 50%, it demonstrated that all included studies were lack of heterogeneity; thus, the Mantel–Haenszel method (fixed effect model) was used to merge the studies, or else the random effect model was adopted. Subgroup analyses were performed for different ethnicity, diagnosis, region, cut-off value, control sources, and so on. In addition, potential publication bias was diagnosed and measured by Funnel plots.

### 2.7 Quality of evidence

We will evaluate the quality of evidence for the outcomes by using the Grading of Recommendations Assessment, Development and Evaluation (GRADE) system.^[15]^ The quality of evidence will be evaluated across the domains of risk of bias, consistency, directness, precision, and publication bias. According to GRADE, the quality of evidence can be rated as high, moderate, low, and very low, which is reflecting the strength of clinical recommendation.

## 3 Discussion

GC is the third most lethal malignancy and the fifth most frequently diagnosed cancer in the worldwide. The high rate of mortality and poor OS of GC illustrated that the primary disease prevention, early diagnosis, and a long time follow-up should be high priorities. It is necessary and crucial to identify predictive and prognostic biomarkers for gastric cancer patients, which can guide better clinical decision regarding treatment and outcomes. The aim of this study was to investigate the association the expression of LncRNA MT1JP and the OS and the clinical characteristics for the GC patients.

## Author contributions

CLZ, JCM, and YQL participated in the study design,and had significant role in development of the selection criteria, risk of bias assessment strategy, and data extraction criteria; CLZ, YBZ, and MXD participated in the study design and interpretation; JCM and YQL contributed to the development of the selection criteria, the risk of bias assessment strategy and data extraction criteria; CLZ and JCM developed the search strategy; CLZ and YBZ participated in the statistical analysis. CLZ, JCM, YQL, and MXD participated in critical review; all authors read, provided feedback, and approved the final manuscript.

**Conceptualization:** Chenglou Zhu, Jichun Ma, Yaoqi Li, Mingxu Da.

**Data curation:** Jichun Ma, Yaoqi Li, Yongbin Zhang.

**Formal analysis:** Chenglou Zhu, Jichun Ma, Yongbin Zhang, Mingxu Da.

**Investigation:** Chenglou Zhu, Yaoqi Li, Yongbin Zhang.

**Methodology:** Jichun Ma, Yaoqi Li, Yongbin Zhang, Mingxu Da.

**Project administration:** Jichun Ma.

**Resources:** Chenglou Zhu, Yongbin Zhang, Mingxu Da.

**Software:** Chenglou Zhu.

**Validation:** Chenglou Zhu.

**Visualization:** Chenglou Zhu, Yaoqi Li, Mingxu Da.

**Writing – original draft:** Chenglou Zhu, Jichun Ma, Yaoqi Li, Yongbin Zhang, Mingxu Da.

**Writing – review & editing:** Chenglou Zhu, Mingxu Da.
